# Medium- to Long-Term Survivorship Analysis Following Copeland Resurfacing Hemiarthroplasty

**DOI:** 10.7759/cureus.95489

**Published:** 2025-10-27

**Authors:** Aliasger Bharmal, Nikhil Gokhale, James Blacknall, Sherri Curtis, Ganesh Prasad, Amit Bidwai, Jomy Kurian

**Affiliations:** 1 Orthopaedics, Sherwood Forest Hospitals NHS Foundation Trust, Mansfield, GBR; 2 Trauma and Orthopaedics, Chesterfield Royal Hospital, Chesterfield, GBR

**Keywords:** copeland, crha, cuff tear arthropathy, hemiarthroplasty, mark iii, osteoarthritis, resurfacing, rheumatoid arthritis, shoulder, survivorship

## Abstract

Background

Copeland resurfacing hemiarthroplasty (CRHA) was developed as a bone-preserving alternative to stemmed hemiarthroplasty, offering advantages such as preservation of native anatomy and quicker recovery. However, there is limited evidence regarding its long-term survivorship.

Objective

The objective of this study is to determine the medium- to long-term survival outcomes and functional results of CRHA performed in a single-surgeon series.

Methods

A retrospective cohort study was conducted on patients who underwent CRHA between 2007 and 2013 at King's Mill Hospital, Mansfield, UK. There were no inclusion or exclusion criteria; all patients had significant morbidity and radiological evidence of glenoid arthropathy (Walch classification). The Oxford Shoulder Score (OSS) was collected pre- and postoperatively. Kaplan-Meier survival analysis was performed. Statistical analysis was carried out using IBM SPSS Statistics for Windows, Version 28 (Released 2021; IBM Corp., Armonk, New York, United States), with significance set at p<0.05.

Results

Eighty CRHAs were performed in 72 patients (eight bilateral procedures). The mean follow-up was 79 ± 18 months (range 50-122), corresponding to approximately 6.5 years. The primary indication was osteoarthritis (76.3%), followed by cuff tear arthropathy (CTA) (16.3%), rheumatoid arthritis (RA) (5%), and post-trauma (1.3%). The mean preoperative OSS was 16 ± 5, which doubled postoperatively to 32 ± 8 (p<0.05). Fifteen patients (18.8%) underwent revision surgery, with a mean time to revision of 49 ± 11 months. Projected survival at five, seven, and 10 years was 83%, 81%, and 79%, respectively.

Conclusion

CRHA improved pain and function in the medium term but demonstrated a higher revision rate compared with other arthroplasty options, particularly in patients with CTA and RA. These findings should guide patient selection and shared decision-making.

## Introduction

Copeland resurfacing hemiarthroplasty (CRHA) is a bone-preserving alternative to stemmed hemiarthroplasty, offering preservation of native anatomy, less invasiveness, and quicker recovery [1-3]. This study evaluates CRHA outcomes in a single-surgeon series, allowing consistent operative technique and postoperative care. The current series uses the Mark III prosthesis, which incorporates hydroxyapatite coating for biological fixation [2,4].

CRHA has been used for osteoarthritis (OA), rheumatoid arthritis (RA), cuff tear arthropathy (CTA), avascular necrosis, and post-infective arthropathies, with the only absolute contraindication being acute fracture [4]. Although short- to medium-term results of CRHA are well reported, there is limited evidence on long-term survivorship. Dekker et al. reported survival of 90% at 5 years and 83% at 10 years in OA patients across multiple surgeons [5].

This study evaluates medium- to long-term outcomes of CRHA with one of the longest mean follow-up periods reported in a single-surgeon series, providing valuable insight into implant survivorship, functional recovery, and revision patterns across different underlying pathologies. Unlike previous multicenter reports, this series offers the advantages of operative consistency, independent outcome assessment, and detailed stratification by diagnosis.

This article was previously presented as a meeting abstract at the British Indian Orthopaedic Annual Scientific Meeting on June 15, 2023 [6].

## Materials and methods

Study design and population

This was a retrospective cohort study including all patients who underwent CRHA performed by the senior author between 2007 and 2013 at King’s Mill Hospital, Mansfield, UK.

There were no additional inclusion or exclusion criteria based on diagnosis, age, or comorbidity. All patients were included, provided they had symptomatic and radiologically confirmed glenohumeral arthropathy (classified according to Walch morphology) with associated functional impairment warranting surgical intervention. This ensured that the cohort represented a complete and consecutive single-surgeon series, minimizing selection bias and enhancing reproducibility.

In total, 72 patients underwent 80 CRHAs, including eight bilateral procedures.

Surgical technique

All patients received a Copeland Mark III implant. Surgery was performed in the beach-chair position through a deltopectoral approach. Subscapularis tenotomy and biceps tenodesis were routinely performed. Undersizing was preferred to avoid overstuffing. Subscapularis repair was completed with non-absorbable sutures, and patients were placed in a shoulder immobilizer postoperatively.

Postoperative management and follow-up

Physiotherapy-led rehabilitation commenced immediately postoperatively, with most patients discharged the following day. Minimum clinical follow-up was 12 months, with longer-term assessments performed in person. Patients were assessed at six weeks, six months, 12 months, and annually thereafter, with the Oxford Shoulder Score (OSS) [7] and radiographs collected at each visit.

In cases of death or missed follow-up, patients were censored at the last available assessment for Kaplan-Meier survival analysis.

Outcomes

Functional outcomes were measured using the OSS, a 12-item patient-reported tool (maximum 48, best function). Pre- and postoperative OSSs were collected by independent physiotherapists. Medical and surgical complications, revision procedures, and indications were recorded. Radiographs were reviewed using the Walch classification [8]. Kaplan-Meier survival analysis was performed using revision for any cause as the endpoint.

Statistical analysis

Continuous variables are presented as mean ± standard deviation. Paired t-tests were used to compare pre- and postoperative OSS. Kaplan-Meier survival analysis was conducted to calculate survivorship at five, seven, and 10 years, with 95% confidence intervals. Patients who died during follow-up without prior revision were censored at the time of death. Missing data were handled using pairwise deletion.

All statistical analyses were performed using IBM SPSS Statistics for Windows, Version 28 (Released 2021; IBM Corp., Armonk, New York, United States), with statistical significance set at p<0.05.

## Results

Eighty CRHAs were performed in 72 patients (65.3% female), with a mean age of 69 ± 11 years (range 46-92). The indications were OA (76.3%), CTA (16.3%), RA (5%), and post-traumatic arthropathy (1.3%). The mean follow-up was 79 ± 18 months (range 50-122). The mean preoperative OSS was 16 ± 5, improving to 32 ± 8 postoperatively (p<0.05). One patient developed a superficial wound infection, successfully treated with antibiotics. One patient experienced transient median nerve dysfunction complicated by venous thromboembolism.

Fifteen patients (18.8%) underwent revision surgery, 11 reverse shoulder arthroplasties (RSAs) and four total shoulder arthroplasties (TSAs), with a mean time to revision of 49 ± 11 months. Projected survival at five, seven, and 10 years was 83%, 81%, and 79%, respectively.

Thirteen patients died during follow-up, corresponding to mortality rates of 4.2% at two years, 8.3% at five years, and 18.1% at seven years. A total of 15 CRHAs required revision (mean 49 ± 11 months post-surgery), primarily for persistent pain and stiffness (93.3%) and periprosthetic fracture (6.7%). As noted above, these comprised 11 RSAs and four TSAs.

The revision rates stratified by preoperative indication are summarized in Table 1.

**Table 1:** Revisions stratified by preoperative indication CRHA: Copeland resurfacing hemiarthroplasty

Preoperative diagnosis	CRHAs performed (n)	Revisions (n)	Revision rate (%)
Osteoarthritis (OA)	61	10	16.4
Cuff tear arthropathy (CTA)	13	4	30.8
Rheumatoid arthritis (RA)	4	1	25.0
Post-traumatic / Unstable posterior dislocation	2	0	0.0
Total	80	15	18.8

Radiographic outcomes

Radiographs were reviewed using the Walch classification [8]. Preoperative Walch morphology was predominantly type A (91.3%) (A1: 32.5%, A2: 58.8%), followed by B2 (7.5%) and C (1.3%). Of the 15 revisions, 26.7% demonstrated progression of humeral head migration consistent with glenoid erosion.

Survivorship

**Figure 1:**
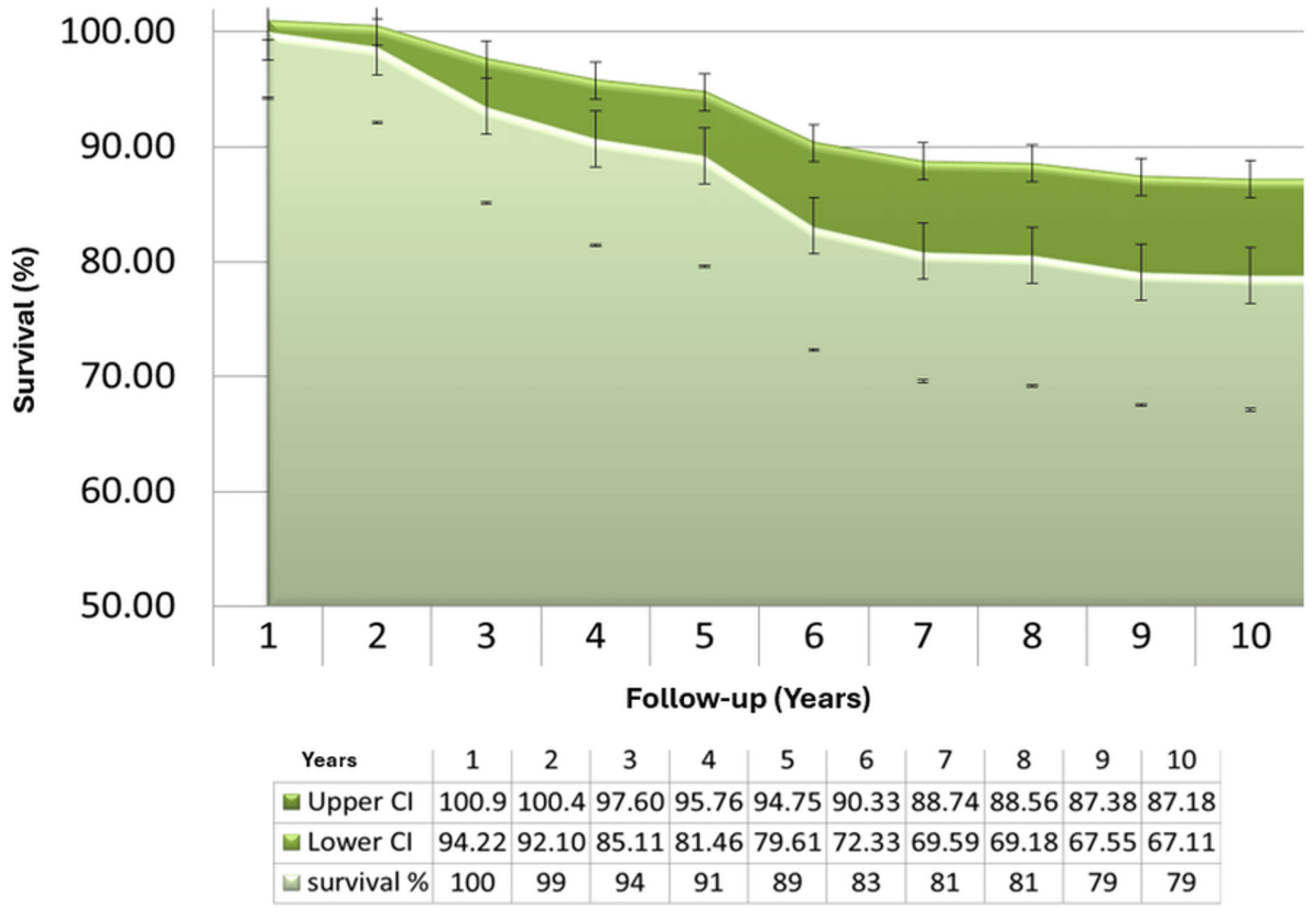
Kaplan-Meier survival graph Kaplan-Meier survival curve throughout our follow-up with a 95% confidence interval

We undertook a Kaplan-Meier survival analysis (Figure 1) to evaluate survivorship at different intervals (five, seven, and 10 years) following CRHA surgery. 

## Discussion

This single-surgeon series demonstrates that CRHA provides meaningful improvement in shoulder function and pain relief at medium- to long-term follow-up. The mean OSS improved from 16 ± 5 preoperatively to 32 ± 8 postoperatively (p<0.05). However, the overall revision rate was relatively high at 18.8%, with the highest rates observed among patients with CTA (30.8%) and RA (25.0%). Kaplan-Meier analysis demonstrated survivorship of 83%, 81%, and 79% at five, seven, and 10 years, respectively, with a clear plateau in failures beyond the fifth year.

The strengths of this study lie in its design as a complete, single-surgeon series with long-term follow-up, ensuring consistency in operative technique, implant selection, and rehabilitation. In contrast to previous multicenter studies, all patients were assessed face-to-face by independent physiotherapists using the OSS, reducing response bias and improving data reliability. Additionally, survivorship and revision analysis were stratified by preoperative diagnosis, providing diagnostic-specific insights that are rarely reported in the literature.

CRHA has been used in a variety of pathologies, with OA being the primary indication. In this cohort, 76.3% of patients had OA, providing a meaningful representation of CRHA outcomes in this setting. Other preoperative diagnoses, including CTA and RA, were also included, and stratification of survivorship by indication revealed important differences. Revision rates were notably higher in CTA (30.8%) and RA (25.0%) compared with OA (16.4%), indicating that poorer survivorship in these non-OA pathologies may contribute to the higher overall revision rate observed in this series compared with other published reports. Based on these findings, CRHA is most suitable for OA patients with preserved glenoid morphology, emphasizing careful patient selection to optimize outcomes.

The overall revision rate was 18.8% (15/80), which increased to 22.4% (15/67) after accounting for patient deaths. This remains higher than alternative arthroplasty options, with prior studies reporting 10-year revision-free survival of 90.2% for TSA and 92.2% for stemmed hemiarthroplasty [9-11]. Randomized controlled trials similarly demonstrate lower revision rates for TSA (≈10%) compared with stemmed hemiarthroplasty (≈31%) [12]. The higher revision rate in CRHA may reflect both true implant failure and the lower surgical threshold for revising resurfacing implants, which are technically easier to convert to RSA or TSA than stemmed implants [13]. Analysis of implant-specific failure modes (e.g., loosening vs pain-driven revision) and post-revision outcomes was not feasible in this dataset, and we acknowledge this as a limitation and an area for future study.

Compared with typical TSA or stemmed hemiarthroplasty cohorts, which often include older patients with advanced glenoid wear, glenoid deformity, or complex rotator cuff pathology, our CRHA cohort was younger (mean age 69 ± 11 years) and predominantly had preserved glenoid morphology (91.3% Walch type A). CRHA was therefore primarily offered as a bone-preserving strategy for patients with less severe glenoid disease, allowing maintenance of native anatomy and easier future revision if required. These findings provide insight into patient selection and may inform decision-making in the current era, where RSA is increasingly used for complex pathology.

In typical TSA cohorts, patients are often older and present with more advanced glenoid wear or deformity, sometimes accompanied by rotator cuff insufficiency. These patients are selected for TSA to restore joint mechanics and provide long-term stability, often at the expense of bone preservation. In contrast, our CRHA cohort consisted of younger patients with predominantly preserved glenoid morphology and intact or minimally compromised rotator cuffs. Consequently, CRHA was primarily offered as a bone-preserving strategy with the potential for easier future revision, which also contributes to differences in functional outcomes and revision rates observed between resurfacing and stemmed or total arthroplasty.

Importantly, revision rates plateaued after five years, suggesting that most failures occur early rather than progressively over the long term. This plateau in revision rates beyond five years likely reflects the early identification and revision of implants that failed due to technical or biological factors, such as glenoid erosion or rotator cuff insufficiency. Once these early failures were addressed, long-term implant stability appeared to remain satisfactory in the surviving cohort.

Functional outcomes in this study demonstrated an increase in the mean OSS from 16 ± 5 preoperatively to 32 ± 8 postoperatively (p<0.05). Although this improvement is lower than reported in some other single-surgeon series (OSS ≈42), the larger sample size and longer follow-up enhance confidence in the durability of these results [14]. Despite modest functional gains and relatively high revision rates, CRHA remains effective in pain relief and improving function for selected patients.

This study is limited by its retrospective and observational design, with no randomization or control group, which may introduce inherent selection and observation biases. Additionally, attrition bias is possible, as 13 patients died during follow-up, and their revision status at the time of death was unknown. The mean patient age of 69 years also limits generalization to younger populations, and radiographic progression was not systematically quantified. Furthermore, implant-specific failure modes and post-revision outcomes were not analyzed. Nonetheless, the study’s strengths include a consistent surgical technique and one of the longest follow-up periods reported for a single-surgeon CRHA series, providing valuable insights for patient counseling and surgical decision-making in the context of modern shoulder arthroplasty.

## Conclusions

CRHA provided satisfactory pain relief and functional improvement in the medium term but demonstrated higher revision rates compared with TSA and stemmed hemiarthroplasty, particularly in patients with CTA and RA. Survival plateaued after five years, indicating that most failures occur early.

Careful patient selection is essential when considering CRHA, reserving it for younger patients with preserved glenoid morphology and good bone stock, where bone-preserving strategies are desired. Despite declining use of CRHA in contemporary practice, these findings remain clinically relevant by highlighting appropriate patient selection criteria, particularly younger, active patients with preserved glenoid morphology, where bone preservation and easier revision pathways are advantageous.

## References

[1] Levy O, Copeland SA (2001). Cementless surface replacement arthroplasty of the shoulder. J Bone Joint Surg.

[2] Copeland S, Funk L, Levy O (2002). (iii) Surface-replacement arthroplasty of the shoulder. Curr Orthop.

[3] Mullett H, Levy O, Raj D, Even T, Abraham R, Copeland SA (2007). Copeland surface replacement of the shoulder. Results of an hydroxyapatite-coated cementless implant in patients over 80 years of age. J Bone Joint Surg Br.

[4] Copeland S (2006). The continuing development of shoulder replacement: "reaching the surface". J Bone Joint Surg Am.

[5] Dekker AP, Joshi N, Morgan M, Espag M, A Tambe A, Clark DI (2020). 6-Year clinical results and survival of Copeland resurfacing hemiarthroplasty of the shoulder in a consecutive series of 279 cases. J Clin Orthop Trauma.

[6] Bharmal A, Gokhale N, Curtis S, Prasad G, Bidwai A, Kurian J (2022). Medium to long-term survivorship analysis following Copeland resurfacing hemiarthroplasty. Orthop Procs.

[7] Dawson J, Rogers K, Fitzpatrick R, Carr A (2009). The Oxford shoulder score revisited. Arch Orthop Trauma Surg.

[8] Walch G, Badet R, Boulahia A, Khoury A (1999). Morphologic study of the glenoid in primary glenohumeral osteoarthritis. J Arthroplasty.

[9] Singh JA, Sperling JW, Cofield RH (2011). Revision surgery following total shoulder arthroplasty: analysis of 2588 shoulders over three decades (1976 to 2008). J Bone Joint Surg Br.

[10] Irlenbusch U, Zenz P, Blatter G, Berth A (2019). Adjustable stemmed shoulder hemiarthroplasty: ten-year results of a prospective multicentre study. Orthop Traumatol Surg Res.

[11] Valencia-Ramon EA, Pasache-Lozano R, Bishop AL, Johnston DG, Trenholm JA (2023). Analysis on revision rates of shoulder arthroplasty at a single referral center in Canada. Semin Arthroplasty.

[12] Sandow MJ, David H, Bentall SJ (2013). Hemiarthroplasty vs total shoulder replacement for rotator cuff intact osteoarthritis: how do they fare after a decade?. J Shoulder Elbow Surg.

[13] Ödquist M, Hallberg K, Rahme H, Salomonsson B, Rosso A (2018). Lower age increases the risk of revision for stemmed and resurfacing shoulder hemi arthroplasty. Acta Orthop.

[14] Al-Hadithy N, Domos P, Sewell MD, Naleem A, Papanna MC, Pandit R (2012). Cementless surface replacement arthroplasty of the shoulder for osteoarthritis: results of fifty Mark III Copeland prosthesis from an independent center with four-year mean follow-up. J Shoulder Elbow Surg.

